# Examining Factor Structure and Validating the Persian Version of the Pregnancy’s Worries and Stress Questionnaire for Pregnant Iranian Women

**DOI:** 10.5539/gjhs.v7n6p308

**Published:** 2015-04-23

**Authors:** Fariba Navidpour, Mahrokh Dolatian, Farideh Yaghmaei, Hamid Alavi Majd, Seyed Saeed Hashemi

**Affiliations:** 1 Department of Midwifery, International Branch, Shahid Beheshti University of Medical Sciences, Tehran, Iran; 2 Assistant professor, Department of Midwifery and Reproductive Health, School of Nursing and Midwifery, Shahid Beheshti University of Medical Sciences, Tehran, Iran; 3 Associate Professor, Department of Nursing Zanjan Branch, Islamic Azad University, Zanjan, Iran; 4 Professor, Department of Biostatistics, School of Paramedical Sciences, Shahid Beheshti University of Medical Sciences, Tehran, Iran; 5 Department of Psychology, Tehran University, Tehran, Iran

**Keywords:** pregnancy worries and stresses, translation, validity, factor structure

## Abstract

**Background and Objectives:**

Pregnant women tend to experience anxiety and stress when faced with the changes to their biology, environment and personal relationships. The identification of these factors and the prevention of their side effects are vital for both mother and fetus. The present study was conducted to validate and to examine the factor structure of the Persian version of the Pregnancy’s Worries and Stress Questionnaire.

**Materials and Methods:**

The 25-item PWSQ was first translated by specialists into Persian. The questionnaire’s validity was determined using face, content, criterion and construct validity and reliability of questionnaire was examined using Cronbach’s alpha. Confirmatory factor analysis was performed in AMOS and SPSS 21. Participants included healthy Iranian pregnant women (8-39 weeks) who refer to selected hospitals for prenatal care. Hospitals included private, social security and university hospitals and selected through the random cluster sampling method.

**Findings:**

The results of validity and reliability assessments of the questionnaire were acceptable. Cronbach’s alpha calculated showed a high internal consistency of 0.89. The confirmatory factor analysis using the χ^2^, CMIN/DF, IFI, CFI, NFI and NNFI indexes showed the 6-factor model to be the best fitted model for explaining the data.

**Conclusion:**

The questionnaire was translated into Persian to examine stress and worry specific to Iranian pregnant women. The psychometric results showed that the questionnaire is suitable for identifying Iranian pregnant women with pregnancy-related stress.

## 1. Introduction

Pregnancy is a pleasant period in women’s life. However, this natural process might be disrupted by internal and external stressors. Most pregnant women experience anxiety and stress in the face of the physical symptoms and the biological and biochemical changes of pregnancy, possible changes to personal and family relationships, social and economic issues, pregnancy-related medical and obstetric complications, newborn safety and stages of labor and delivery (Dunkel Schetter, 2011).

Stress refers to a situation in which internal and external conditions weaken or disrupt the normal functioning of humans’ energy and soul and consequently stimulate the autonomic nervous system, in particular the sympathetic nervous system, which ultimately create anxiety in the individual (B. J. Sadock & V. A. Sadock, 2008; Tordjman, Gragnier-Deferre, & Graignic, 2014). Pregnancy and childbirth form a specifically stressful situation that only women experience, which makes the prevalence of anxiety -a common complication of stress- twice as much in women than in men (Kaplan & Sadock, 2007). Different studies have been conducted in different societies to identify stressors in pregnant women and their outcomes for pregnancy, leading to the publication of various statistics. The studies conducted on the prevalence of stress in early pregnancy in different societies found that 16.5-74% of women suffer from stress during their early pregnancy (Carolan-Olah & Barry, 2014; Rubertsson, Hellstrom, Cross & Sydsjo, 2014). Findings of a few studies conducted on this subject in Iran showed the prevalence of stress and anxiety in pregnant women to be 16.7% and 49%, respectively (Alipour, Lamyian, & Hajizadeh, 2012; Salari, Firoozi & Sahebi, 2005).

Perceived stress causes biological responses in the body that are followed by some symptoms. Anxiety is an important complication of stress. It is an unpleasant feeling such as fear and worry spread and accompanied by one or more physical symptoms, such as headache, palpitations, mild abdominal pain, dyspnea, perspiration, restlessness and sometimes pallor (B. J. Sadock & V. A. Sadock, 2008). The normal activity of the HPA (hypothalamus, pituitary, adrenal) axis is the principle biological consequences of perceived stress that eventually leads to the release of cortisol (Sandman, Davis, & Glynn, 2012). The cortisol released from the HPA system, increases the production of cortisol through its positive feedback effect on the placental CRH, despite the negative feedback effect of the HPA axis on the CRH (Sandman et al., 2012). Moreover, the sympathetic nervous system is stimulated in response to stress and therefore releases catecholamines, including epinephrine and norepinephrin (Kaplan & Sadock, 2007). The irreversible complications of the activation of these hormone systems in women include the increased severity of pregnancy nausea and vomiting and exacerbated cardiovascular diseases and blood pressure (Hobel, Goldstein, & Barrett, 2008). Furthermore, the hormones released due to stress and anxiety have significant consequences, including preterm childbirth, fetal weight loss and impaired fetal development and growth (Graignic-Philippe, Dayan, Chokron, Jacquet & Tordjman, 2014). Given the importance of these complications, numerous studies have been conducted with the purpose of identifying pregnancy stressors. Arch (2013) conducted a study seeking to find the reasons for pregnant American women’s tendency toward drinking and used the PWSQ, that is, the 10-item brief version of the PRAQ (PRAQ-revised)(Huizink, Mulder, Robles de Medina, Visser & Buitelaar, 2004) in addition to personal, family and economic factors –a few of which were included in the original PRAQ (van den Berg’s 58-item questionnaire (Van den Bergh, 1990), but which Huizink et al. (2004) had not taken account of in their study.

Given that no comprehensive and applicable questionnaires (questionnaire that is both psychometrically sound yet and as brief as possible) have ever been used for assessment of pregnancy’s stress in Iranian pregnant women, the researcher seeks to examine the psychometrics of Arch’s 25-item PWSQ in Iran.

## 2. Materials and Methods

The present psychometric (LoBiondo-Wood, Haber, Berry & Yost, 2013) methodological research was conducted in 2014.

### 2.1 Stage 1-Translation of Questionnaire

The researcher translated the questionnaire into Persian through the 3-step method after obtaining the designer’s permission (Acquadro, Lafortune, & Mear, 2003; Leinonen, Leino-Kilpi, Ståhlberg, & Lertola, 2001). To carry out the 3 steps, the questionnaire was translated into Persian separately by two; then, Persian questionnaire was translated into English again by another English and Persian language expert; eventually, a committee of team members prepared the final Persian version of the questionnaire according to the rules of semantic, terminological, experiential and comprehensive parity.

Arch’s PWSQ consists of 6 subcategories: childbirth and the experience of motherhood (4 items), newborn safety (5 items), mother and newborn preferences (2 items), personal and family issues (3 items), mother’s health (6 items) and personal and job-related issues (5 items). Each questionnaire item measures with the Likert scale from 1 to 5 (Never = 0, rarely = 1, sometimes = 2, often = 3 and always = 4).

### 2.2 Stage 2- Psychometric Analysis of Instruments

This step was dedicated to evaluation of the psychometric properties of the questionnaire. The present study examined the validity and reliability of PWSQ (Schneider, 2013) For determining the questionnaire’s validity, the face, content, criterion and construct validity were examined and the internal consistency and stability were examined to determine its reliability.

#### 2.2.1 Face Validity

Face validity has both qualitative and quantitative aspects. In this study face validity was examined through both qualitative and quantitative approach (Wright & Stone, 1999). Face validity emphasizes the viewpoints of both the target group (Polit, Beck, & Hungler, 2006) and the experts and the present study also used the viewpoints of both the target group and the experts (Hajizadeh & Asghari, 2012; Netemeyer, Bearden & Sharma, 2003; Shultz, Whitney & Zickar, 2013)To examine the face validity through the qualitative approach, 10 pregnant women (Wilson, 1985) admitted to a private hospital in Tehran were randomly selected and interviewed face to face about the questionnaire items. This part examined the levels of difficulty, irrelevance and ambiguity.

To eliminate inappropriate items in the next step, the importance of each item was determined using the quantitative item score impact method. This step used the viewpoints of the target group that had participated in the qualitative step and also of the expert group consisting of 13 members (2 psychologists with PhDs, 2 clinical psychiatrists, 1 nurse with a PhD, 1 family counselor with a PhD, 4 reproductive health doctors, 1 psychometrist and 2 professors of midwifery). The item score impact was calculated using the following equation: Item Score Impact= Frequency (%) × Importance. Each item was scored within the 5-point Likert scale (Not important at all = 1 to absolutely important =5) by both the participants (the pregnant women) and the experts. The score impact was calculated for each item. Items with a score of 1.5 or higher were retained and deemed appropriate to enter the next step of analysis (Hajizadeh & Asghari, 2012; Lacasse, Godbout & Series, 2002)

#### 2.2.2 Content Validity

Content validity was also examined through both qualitative and quantitative approach. The present study determined the qualitative content validity using the experts’ opinions (Wilson, 1985). The experts first performed a qualitative examination of the questionnaire based on the rules of grammar, wording, item allocation and proper scaling and then presented their feedback.

For the quantitative examination of the content validity, two indexes Content Validity Ratio (CVR) and Content Validity Index (CVI) were used. To calculate the CVR, the 13 experts were requested to score each of the 25 items of the questionnaire on the basis of a 3-point Likert scale (It is necessary=1, It is useful but not necessary=2, It is not necessary=3). If the calculated CVR was larger than its corresponding value in Lawshe’s table (based on the assessments of the 13 experts), i.e. larger than 0.54, that item was retained in the questionnaire with the statistical significance level of P<0.05 (Lawshe, 1975). Then, CVI was determined base on Waltz and Bausell content validity index (Waltz & Bausell 1983). The same group of experts expressed their opinions about the relevance, simplicity and comprehensibility of each item of questionnaire in a 4-point Likert scale. If an item’s calculated CVI was larger than 0.79, it was deemed appropriate; if, however, it was between 0.7 and 0.79, the item was questionable and required revision; and if the CVI was lower than 0.7, the item had to be removed entirely (Polit, Beck, & Owen 2007). If the average CVI for the entire questionnaire was equal to or higher than 90%, then the S-CVI/Ave and consequently the scale were acceptable (Polit, Beck, & Hungler, 2006) (Flowchart).

#### 2.2.3 Criterion Validity

The criterion validity was examined using 2 instruments. The first instrument was State-Trait Anxiety Inventory (STAI) (Spielberger, Gorsuch, & Lushene, 1970). This inventory had already been standardized in Iran (Mahram, 2002) and consist of two parts for measuring State and Trait anxieties. The part 1 is related to State Anxiety and consists of 20 items that assess individual’s feelings at the moment of responding. The part 2 is related to Trait Anxiety and consists of 20 items that assess individual’s general state and feelings. The 2 scales (PWSQ and STAI) were used to measure 50 randomly-selected pregnant women and were then compared, indicating a favorable correlation, which also confirmed the predictive validity of the questionnaire (Flowchart).

The second instrument used to examine the criterion validity was the researcher-made questionnaire of the Stressors Associated with Pregnancy designed by Salari et al. (1998). This questionnaire consists of 51 items within 6 domains, including health (24 items), others’ perception of oneself (6 items), religion (5 items), finance (3 items), environment (7 items) and the personal and family domain (6 items). The inventory was selected for assessing the criterion validity because it examines stressors in pregnant women. The 2 instruments were used to measure 50 pregnant women and were then compared, indicating a favorable correlation and also confirming the concurrent validity.

#### 2.2.4 Construct Validity

Given that the PRAQ items were determined, the confirmatory factor analysis was used to confirm the model for use among the Iranian population. Different examinations of sample size showed that 500 subjects were required for the confirmatory factor analysis (Munro, 2013); however, taking account of a 10% sample loss, 550 pregnant women were finally selected for the study.

The samples were selected from prenatal care units of private, university and social security hospitals in Tehran through the stratified cluster sampling method. Therefore, Tehran was divided into 5 regions of north, south, east, west and center. A hospital was randomly selected from each region. Then, 550 pregnant women were randomly selected to enter the study based on the statistical information available and the number of pregnant women admitted to each hospital. Finally, a total of 502 pregnant women had fully completed the questionnaire. The inclusion criteria consisted of being Iranian, being aged 18-45, being at the gestational age of 8-39 weeks, not having experienced any important life-events other than pregnancy during the past 6 months, not having children with a disease or with physical and mental disabilities, having a singleton embryo and a normal course of pregnancy (Without every complication that is lead to hospitalization) and living with their husband at the time of the study.

The data were examined for the confirmatory factor analysis in SPSS-21 and AMOS. Given the AMOS output consist of Chi-square (χ^2^) test, Chi-square/Degree of Freedom Ratio (Normalized chi-square CMIN/DF), Comparative Fit Index (CFI), Incremental Fit Index (IFI), Tucker-Lewis Index or the Non-Normalized Fit Index (NNFI), Bentler-Bonett Index or Normalized Fit Index (NFI), and Root Mean Square Error of Approximation (RMSEA) were used for Confirmatory Factor Analysis (Ghasemi, 2013).

#### 2.2.5 Questionnaire’s Reliability

The questionnaire’s reliability was determined through the internal consistency and the stability measures. Cronbach’s alpha is a famous method for measuring internal consistency (LoBiondo-Wood et al., 2013; Polit et al., 2006). In this respect, 50 pregnant women randomly selected from the selected hospitals and the questionnaires were filled out by them. The internal consistency of questionnaire (Cronbach’s alpha) was determined. The internal consistency was acceptable if the Cronbach’s alpha measurement was equal to or greater than 0.70.

For assessing of stability of PWSQ, Test re Test method was examined. In this respect 30 pregnant women randomly selected from one of the selected hospitals, who then filled out the questionnaire over 2 stages with a 14-day interval (Anastasi, 1990). The obtained scores were then compared with each other using Pearson’s correlation coefficient. An optimum correlation coefficient is higher than 0.70 (Munro, 2013).

In order to respect the ethical considerations, the study was conducted upon receiving the consent of the presidents of Shahid Beheshti University, University of Tehran, Social Security Organization and the hospital directors. Participants were given all the necessary information prior to participation in the study and were also ensured of the confidentiality of their information and they could withdraw from the study whenever they liked. Then, participants gave their informed verbal consent to the researcher before they participated in the study.

## 3. Findings

In this study, 550 pregnant women participated for the confirmatory factor analysis who 502 filled out the questionnaire in full. Table 1 shows participants’ details (with a mean age of 29.27±8.9 years).

**Table 1:** Frequency distribution of age group, education, occupation and mother’s parity in 5 region of Tehran

Mother’s specification	Name of group	Frequency
Age	18-30	143-%28
	30-35	310-%61.6
	35-40	41-%8.6
	>40	8-%1.8
Level of education	6-%1.2	Illiteracy
	primary school	45-%9.0
	middle school	71-%14.1
	high school	35-%7.0
	diploma	203-%40.4
	technician	62-%12.4
	bachelor of science	73-%14.5
	master of science	6-%1.2
	PhD	1-%.2
Gestational age	8-12 week	25-%5.0
	12-28 week	138-%27.5
	28-39 week	339-%67.5
Occupational status	housewife	421-%83.9
	worker	27-%5.4
	employee	45-%9.1
	others	9-%1.6
Number of pregnancy	1	230-%45.7
	2	174-%34.6
	3	76-%15.1
	4	16-%3.2
	5	5-%1.2
	6	1-%.2

**Face Validity:** Assessment of the qualitative face validity confirmed all the 25 items. In the assessment of the quantitative face validity, the impact item score for both the target and the expert groups was higher than 1.5 and no single item was therefore eliminated.

**Content Validity:** Assessment of the qualitative content validity confirmed the items in accordance with the expert viewpoints. In the assessment of the quantitative content validity, the CVR of all the items was between 0.55 and 1 and no item was therefore removed. As for the CVI, the items’ average values based on relevance, comprehensibility, simplicity and S-CVI/Ave were calculated as 0.92, 0.98, 0.98 and 0.94, respectively. No items were removed in this step as all of them were deemed appropriate for inclusion in the rest of the stages of the psychometric procedure.

**Criterion Validity:** In the assessment of the criterion validity, after calculating the PRAQ score and State-Trait Anxiety Inventory scores, the correlation between the state-trait anxiety inventory and the entire instrument were obtained as r=0.70 and P=0.000, r=0.69 and P=0.000, and r=0.72 and P=0.000.

The correlation between the research instrument and questionnaire of the Stressors Associated with Pregnancy was acceptable (r=0.72 and p=0.000).

**Construct Validity:** The results of the confirmatory factor analysis confirmed the model and the questionnaire items showed favorable fit indexes:

CMIN = 760/750; CMIN/DF = 3/170; RMSEA = 0.6; and CFI = 0.88

**Reliability:** The internal consistency and stability of the questionnaire determined the reliability. Internal consistency of the questionnaire was examined by Cronbach’s alpha and was calculated as 0.737, 0.713, 0.757, 0.715, 0.79, 0.823 and 0.891 for items 1, 2, 3, 4, 5 and 6 and also for the entire questionnaire. The stability of the questionnaire was examined through the test-retest method and was deemed favorable (r=0.98 and p=0.000).

**Figure:**
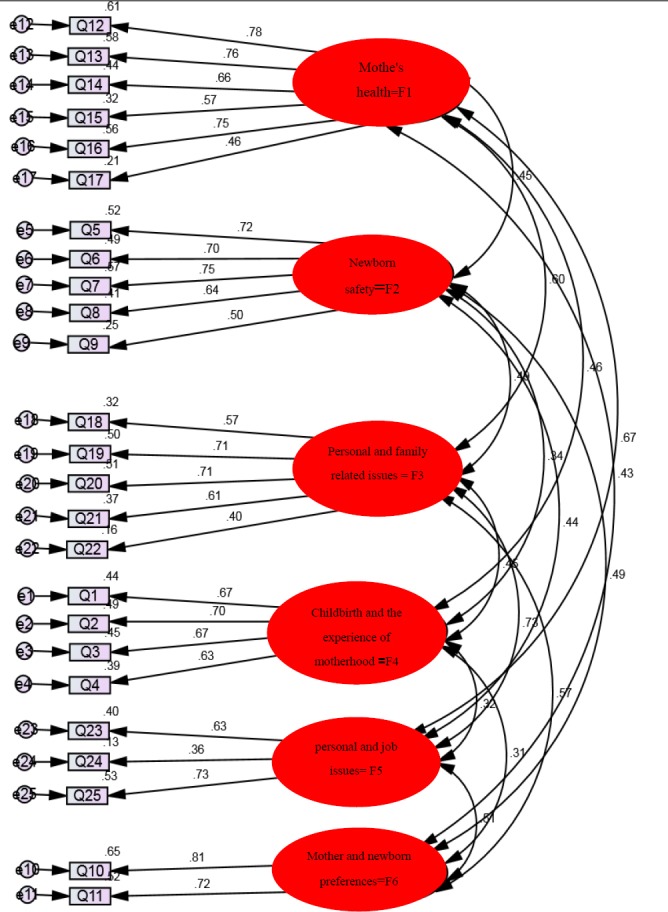
The output model for the PWSQ factors in AMOS

**Table 2:** Confirmatory Factor Analysis fit indexes for the 6-factor model in AMOS

RMSEA	CFI	NFI	P	IFI	NNFI	Χ^2^	DF	CMN/DF
0.06	0.88	0.85	0.000	0.88	0.89	760.750	240	3.1

**Figure:**
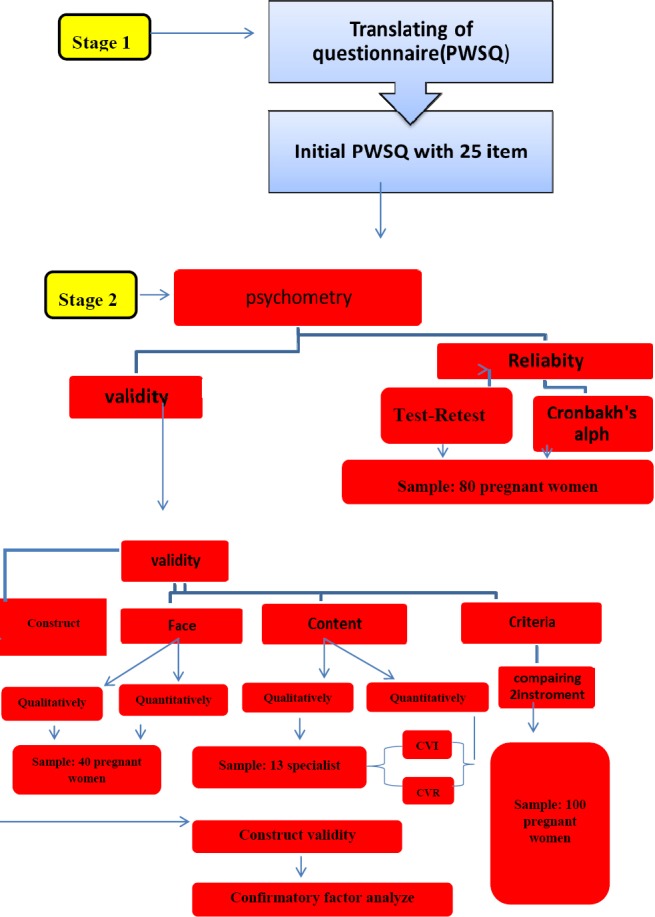
Flowchart

## 4. Discussion

Uncontrolled stress and concern, and their major complication, anxiety, affect the health of both mother and fetus. The early identification of stress in pregnant women and providing the necessary instructions can bring women a pleasant experience of pregnancy. An instrument with a high validity and reliability is therefore crucial for this purpose (Cha & Masho, 2013).

The present study showed that, with its favorable psychometric results, the PWSQ is adequately capable of assessing and being used among the population of Iranian pregnant women. The face and content validity of the questionnaire confirmed the simplicity and comprehensibility. The CVI proved the relevance of the items and showed a considerable degree of agreement (mean=94.61) based on the expert viewpoints.

The reliability of the questionnaire was determined by high Cronbach’s alpha and the Test-Retest method. As shown before, there was a significant relationship between the questionnaire’s factors and the entire instrument, which indicates that pregnancy-related stress and tension are greatly related to stress about childbirth, newborn’s health, mother’s health, personal and family issues and social and economic problems. Arch, who had also used Cronbach’s alpha, showed through his regression model that some social and family factors are good predictors of pregnancy-related stress (Arch, 2013).

In determining the criterion validity, the correlation between the State-Trait Anxiety Inventory (STAI) and the PWSQ showed that the PWSQ can be a predictor of anxiety in pregnant women. Huizink et al. (Huizink et al., 2002; Huizink et al., 2004) used the convergent validity to examine the correlation of the 34-item and the 10-item PRAQs with the Perceived Stress Scale (PSS) and the Daily Hassles Scale (DHS). They found the correlation between the two instruments to be favorable and took it as the questionnaire’s predictive validity, as well.

Moreover, the correlation between the scores obtained for the PWSQ and Salari’s questionnaire shows that although both instruments measure stress and anxiety, the PWSQ is shorter and more practical as all psychometric stages have been performed for it.

The 6-factor construct was confirmed by the present study and through the confirmatory factor analysis for use among the population of Iranian pregnant women. This study used 6 different fit indexes of the comparative and the parsimony normed type for its confirmatory factor analysis. In the study conducted by Huizink et al. (2004) on Dutch women from the Netherlands, the results of the confirmatory factor analysis of the 34-item PRAQ that consisted of such factors like fear of changing in the relationship with the spouse, fear of changing in temperament and fear of the relationship with the newborn and growing to like him, showed that the comparative fit indexes were not acceptable and also had a high error variance. However, the results of the confirmatory factor analysis of Arch’s PWSQ (2013) that included the aforementioned factors showed acceptable fit indexes for the Iranian population. In Arch’s study (2013), factors such as income expressed the 49% parity variance of pregnancy anxiety, which was similar the results of the present study. Main feature of the present study is that it performed all the stages of the psychometric procedure and standardization for the instrument. Previous studies that had used different versions of the PRAQ and other instruments to identify stress in pregnant women rarely performed all the stages of the psychometric procedure or had a sufficient sample size. Taking account of theoretical models in designing questionnaires and their standardization increase their reliability for assessing pregnancy-related stress and leads to the performance of necessary interventions for reducing stress (Alderdice, Lynn, & Lobel, 2012; Cha & Masho, 2013)

## 5. Conclusion

The Pregnancy’s Worries and Stress Questionnaire was translated into Persian to examine stress and worry specific to Iranian pregnant women. The psychometric results (validity and reliability) showed that the questionnaire is suitable and reliable for identifying stress and anxiety in pregnant women and also the questionnaire can be used among this vulnerable group of Iranians, i.e. pregnant women.

## Appendix

**Table:** Pregnanacy’s Worries and Stress Questionnaire

subscale	PREGNANACY’S WORRIES AND STRESS QUESTIONNAIRE					
Childbirth and mother experience of motherhood	1. I am worried about the pain of contractions and the pain during delivery.	Always=4	Often=3	Sometimes=2	Rarely=1	Never=0
	2. I am anxious about the delivery because I have never experienced one before.					
	3. I am worried about not being able to control myself during labor and fear that I will scream.					
	4. I am worried that I won’t know what to do as a new mom.					
Newborn safety	5. I am afraid our baby will be stillborn, or will die during or immediately after delivery.					
	6. I am afraid that our baby will suffer from a physical defect or worry that something will be physically wrong with the baby					
	7. I sometimes think that our child will be in poor health or will be prone to illnesses					
	8. I am worried about whether I might do something to harm my baby, intentionally or unintentionally.					
	9. I am worried about how to cope when my baby is hard to soothe					
Mother and newborn preferenc	10. I am worried that my baby won’t love me.					
	11. I am worried that I won’t love my baby.					
Mother’s health	12. I am worried about the fact that I shall not regain my figure after delivery.					
	13. I am worried that I am unattractive.					
	14. I am worried about my weight.					
	15. I am worried about not getting enough sleep.					
	16. I am worried about body changes due to pregnancy and labor/delivery.					
	17. I am worried about discomforts of pregnancy (such as heartburn, incontinence, back pain).					
Personal-family	18. I am worried that I won’t be able to help my baby in the way that he/she will need.					
	19. I am worried that I won’t do a good job as a new mom.					
	20. I am worried about whether I will be a good mother.					
	I am worried about physical intimacy with my partner/husband.					
	22. I am worried about finances.					
Personal-job	23. I am worried about being a stay at home mom.					
	24. I am worried about balancing my work and home life.					
	25. I am worried about whether my relationship with my partner/husband will be challenged.					
score						
